# Moral distress-associated sociodemographic and occupational aspects in nursing managers at federal university hospitals^*^

**DOI:** 10.1590/1980-220X-REEUSP-2021-0447en

**Published:** 2022-05-20

**Authors:** Michel Maximiano Faraco, Francine Lima Gelbcke, Laura Cavalcanti de Farias Brehmer, Flávia Regina Souza Ramos, Dulcinéia Ghizoni Schneider

**Affiliations:** 1 Universidade Federal de Santa Catarina, Hospital Universitário Professor Polydoro Ernani de São Thiago, Florianópolis, SC, Brazil.; 2 Universidade Federal de Santa Catarina, Programa de Pós-Graduação em Enfermagem, Florianópolis, SC, Brazil.; 3 Universidade Federal de Santa Catarina, Programa de Pós-Graduação em Gestão do Cuidado em Enfermagem, Modalidade Profissional, Florianópolis, SC, Brazil.

**Keywords:** Hospitals, University, Health Manager, Stress, Psychological, Ethics, Nursing, Hospitales Universitários, Gestor de Salud, Estrés Psicológico, Ética em enfermería, Hospitais Universitários, Gestor de Saúde, Estresse psicológico, Ética em enfermagem

## Abstract

**Objective:**

To analyze the association between sociodemographic and occupational characteristics and the predictors of Moral Distress in nursing managers of Federal University Hospitals.

**Method:**

Cross-sectional study carried out with 126 nurses. Data were collected online between September 2019 and May 2020 applying the Brazilian Scale of Moral Distress in Nurses. The variables were analyzed using descriptive and bivariate statistics to compare the instrument mean responses in relation to sociodemographic and occupational characteristics (hospital size, region, age, gender, training and experience variables, employment relationships, and workload).

**Results:**

The highest levels of Moral Distress were experienced by nurses in large hospitals, with statistical significance among civil servants with job stability who have no management training, with less time of professional experience and with the highest weekly workload, with emphasis on predictive factors of “safe and qualified care”, “work conditions” and “work team”.

**Conclusion:**

Based on the above, it is understood that studies of this nature allow the generation of adaptive strategies to reduce the impacts of Moral Distress.

## RESUMO

**Objetivo:**

Analisar a associação entre as características sociodemográficas e laborais e os fatores preditores de Distresse Moral em gestores de enfermagem de Hospitais Universitários Federais.

**Método:**

Estudo transversal realizado com 126 enfermeiros. Os dados foram coletados entre os meses de setembro de 2019 e maio de 2020, via online, aplicando-se a Escala Brasileira de Distresse Moral em Enfermeiros. As variáveis foram analisadas por estatística descritiva e bivariada para comparar as médias de respostas do instrumento em relação às características sociodemográficas e laborais (porte do hospital, região, idade, sexo, variáveis de formação e experiências, vínculos e carga horária).

**Resultados:**

Os níveis mais elevados de Distresse Moral foram vivenciados por enfermeiros em hospitais de grande porte, com significância estatística entre os estatutários, sem formação em gestão, com menor tempo de experiência profissional e com a maior carga horária de trabalho semanal, com ênfase nos fatores preditores de “cuidado seguro e qualificado”, “condições de trabalho” e “equipe de trabalho”.

**Conclusão:**

A partir do exposto, entende-se que estudos dessa natureza permitem gerar estratégias adaptativas para reduzir os impactos do Distresse Moral.

## RESUMEN

**Objetivo:**

Analizar la asociación entre las características sociodemográficas y laborales y los factores predictores de Distrés Moral en gestores de enfermería de Hospitales Universitarios Federales.

**Método:**

Estudio transversal realizado con 126 enfermeros. Los datos fueron recolectados entre los meses de septiembre de 2019 y mayo de 2020, a través de la red, se aplicó la Escala Brasileña de Distrés Moral en Enfermeros. Las variables fueron analizadas por estadística descriptiva y bivariada para comparar las medias de respuestas del instrumento en relación a las características sociodemográficas y laborales (porte del hospital, región, edad, sexo, variables de formación y experiencias, vínculos y carga horaria).

**Resultados:**

Los niveles más elevados de Distrés Moral fueron vivenciados por enfermeros en hospitales de gran porte, con significancia estadística entre los funcionarios públicos con estabilidad, sin formación en gestión, con menos tiempo de experiencia profesional y con la carga horaria más extensa de trabajo semanal, con énfasis en los factores predictores de “cuidado seguro y cualificado”, “condiciones de trabajo” y “equipo de trabajo”.

**Conclusión:**

Así siendo, se entiende que estudios con este abordaje permiten generar estrategias adaptativas para reducir los impactos del Distrés Moral.

## INTRODUCTION

Moral Distress (MD) is a phenomenon described in the experiences of health professionals, especially nurses, in numerous health environments, multiple organizational levels, and professional roles. It is expressed through physical and emotional manifestations, arising from a process of recognition and perception of morally conflicting situations, and the awareness of the morally correct action that, due to institutional or social impediments, is not carried out, threatening fundamental values and moral integrity^(1,2)^.

From the point of view of the process, MD is an ethical- moral experience that considers the moral problem as the starting point, requiring some level of (moral) sensitivity, motivated by restlessness and uncertainty, to act according to its moral judgment^(1)^. In the organizational plan, MD can jeopardize the quality of care, patient safety, and lead to an increase in staff turnover^(3,4)^. In care and management, nurses experience difficulties such as excessive workloads with reduced autonomy to manage them, which constitute risk factors for Moral Distress^(5,6)^.

In the context of Federal University Hospitals (*HUF*), nursing managers can experience numerous challenges, usually related to unfavorable working conditions, conflicts within the team, harassment and lack of autonomy, fragmentation of care, and inadequate physical structure, leading to the need for ‘renormatizations’ in the nursing work process organization^(7,8)^.

In this reality, it is perceptible and desirable that nursing managers develop skills and have sufficient experience and training to deal with management challenges and moral problems. Professionals need to develop different skills to exercise moral agency in response to MD, with the ability to understand goals and responsibilities, recognize critical situations and act when ethical action is necessary^(2)^.

The extent to which a situation leads to MD does not seem to be clearly elucidated. There are still few national and international studies about associations between the phenomenon and sociodemographic and occupational variables, for example. Cross-sectional studies and different measurement instruments adapted and validated for particular contexts, whether in practice or demographic environments, predominate^(3,5,6,8)^. This way, the need for studies aiming to minimize these gaps, as well as in- depth studies on methodological alternatives, is noted. Nurse managers have work characteristics that deserve attention, especially those related to leadership and the management model, which can affect the way MD unfolds.

Thus, this study aims to analyze the association between sociodemographic and occupational characteristics and MD predictors in *HUF* nursing managers related to the Brazilian Hospital Services Company – Ebserh. The intention is to support nursing managers in the search for a reflective understanding of the moral agent, as well as to provide an overview of the best possibilities for coping with MD, based on the professional profile.

## METHOD

### Design of Study

This is a quantitative, non-probabilistic study with a cross- sectional design, and convenience sampling.

### Local and Population

It was carried out in 30 Federal University Hospitals related to Ebserh. In the governance structure, the Nursing Division (ND) is the highest level of Nursing Services, there is one Division per hospital. In the other hierarchical lines are the Nursing Managers (NM), as Unit heads or leaders. Nurses indicated for leadership (or interim) positions or in the leadership role of any Nursing Service participated.

### Sample

The total sample size calculated using Effect Size was 118 participants (ND = 30 and NM = 88), and was supported by the software WINPEPI (version 11.65), considering a power of 80%, significance level of 5%, and the ratio of three “NM” participants for each “ND” (3:1).

The process for ND recruitment was supported by the Education and Research Managements of the institutions and/or by direct contact, via email, telephone or texting application, while the ND supported the recruitment of the NM. Data collection took place between September 2019 and May 2020, with the participation of 126 nurse managers (ND = 32 and NM = 94) related to 30 *HUF*s/Ebserh.

### Data Collection

Data collection took place between September 2019 and May 2020. Data were collected online (electronic form), with the Brazilian Scale of Moral Distress in Nurses (MDSN-Br)^(9)^ being applied. This scale has 49 questions indicating the predictive situations of MD in a double Likert scale from 0 to 6, to measure frequency and intensity (0 = never, to 6 = very frequent; and 0 = none, to 06 = very intense; respectively). To analyze the mean scores for MD frequency and intensity, the following ranges were used as parameters: low (0–1.99), moderate (2.00–3.99), and high (4.00–6.00)^(10)^. In this study, MSDN-Br presented a satisfactory level of reliability, with Cronbach’s alpha of 0.976 and between 0.950 and 0.852 for the factors.

The sociodemographic and occupational variables for the purpose of this study were: hospital size, region, age, sex, time since graduation, complementary training, training in the management area, time of work as nurse, time of experience in management, time of experience in the function, number of bonds, type of bond, role, and weekly workload.

### Data Analysis and Treatment

General MD (GMD) was calculated in two moments: frequency score and intensity score are multiplied (FxI) for each of the 49 questions (each item ranging from 0 to 36); – GMD score obtained by adding the “FxI” score, resulting in a scale from 0 to 1,764 (the higher the score, the greater the MD experienced).

The predictors in the MDSN-Br are organized in six factors: “Recognition, power, and personal identity” (F1); “Safe and qualified care” (F2); “Defense of values and rights” (F3); “Working conditions” (F4); “Ethical infractions” (F5); and, “Work teams” (F6). For the purposes of analyzing GDM scores by factor, the following ranges were used as parameters: F1 (0-396), F2 (0-396), F3 (0-288), F4 (0-216), F5 (0-216) and F6 (0-252).

Following coding and categorization, data were tabulated in an electronic spreadsheet and analyzed in the software IBM Statistical Package for Social Sciences (SPSS), version 25.0. Categorical variables are presented in their absolute (n) and relative (%) frequencies.

According to the results of the Shapiro-Wilk normality test, the independent t test or the analysis of variance (depending on the number of categories of the variables studied) was used to compare the averages of the frequency and intensity responses to the instrument items and to evaluate the effect of sociodemographic and occupational characteristics, adopting a significance level of 0.05.

### Ethical Aspects

The project was approved by the Ethics Committee for Research with Human Beings of the proposing institution, under Opinion 3.549.474, in 2019, in compliance with Resolution No. 466/2012 of the National Health Council, with the presence of no conflict of interest. All participants signed the Free and Informed Consent Form.

## RESULTS

Participants had the following predominant characteristics: female (n = 116; 92.1%) aged between 20 and 39 years (n = 72; 57.1%), graduated between 11 and 15 years before (n = 42; 33, 3%); working as a nurse between 11 and 15 years (n = 44; 34.9%); exercising management activity for at least five years (n = 66; 52.4%), the same period in which they hold the nursing leadership position at the HUF/Ebserh (n = 98; 77.8%); having a graduate certificate/residence (n = 59; 46.8%), but with no training in the area of management (n = 70; 55.6%); having job stability (n = 74; 58.7%), single job (n = 96; 76.2%), and working an average of up to 40 hours a week (n = 92; 73%); most of them in medium-sized hospitals with 200 to 399 beds (n = 55; 43.7%), in the Northeast region (n = 52; 41.3%).

It is observed that the average of MD in factors F1, F3 and F5 was higher in younger managers (younger age) when compared with the averages of nurses at older age. There were no significant differences between MD averages in relation to sex. Regarding the Brazilian Region, *HUF* location, only factor F4 (Working conditions) was not significant (p = 0.062). The highest average of MD was in the Southeast region and the lowest in the North region.

The variable “*HUF* Size” showed statistical significance in all factors, with the greater the size of the hospital, the greater the MD experienced by managers. Managers with less time since graduation had a higher average of MD when compared to those with more than 20 years in the profession, specifically in factors F1, F3 and F5. MD average in factor F5 has a significant difference between the categories in terms of time of work as a nurse. In this factor, managers with 11 to 15 years of experience as nurses had a higher average of MD when compared to those with more than 16 years of experience. A similar result was shown regarding the time of management experience, with significant differences in the factors F1, F3 and F5, which present, among the averages, with greater intensities of MD in managers with less experience when compared to those with more than 16 years of management. In all MD factors, it is observed that nurses who do not have training in the management area have the highest MD averages. Regarding the type of relationship of the nursing manager, the average of factors F1, F2, F4 and F6 were significant. The servant with job stability expressed higher averages of MD when compared to the CLT-hired ones (no job stability). Nursing managers who work more than 40 hours a week had higher MD averages, especially in factors F2 and F6, with significance. Based on the calculated averages, it appears that the highest MD scores were indicated among participants located in large *HUFs*, in the Southeast region, by nurses with master’s degree, job stability, up to 49 years of age, with 11 to 15 years of training and experience in management, with at least 5 years in the role as a manager, with no training in the management area and working more than 40 hours a week (Table 1).

**Table 1.** Analysis of Moral Distress among nurse managers and their characteristics, HUF/Ebserh, Sep/2019–May/2020.

Variables	n	Factor 1	Factor 2	Factor 3	Factor 4	Factor 5	Factor 6	∑ F*I
		Mean (SD)	Mean (SD)	Mean (SD)	Mean (SD)	Mean (SD)	Mean (SD)	Mean (SD)
**HUF size**								
Small	54	111.70a (89.18)	131.02a (94.78)	46.19a (61.22)	72.37a (55.26)	52.61a (52.60)	101.76a (59.26)	515.65a (338.43)
Medium	55	148.71a (86.53)	185.07b (85.07)	89.45b (73.67)	94.40a (51.19)	67.89a (44.50)	116.84a (45.22)	702.36b (307.98)
Large	17	247.88b (70.45)	265.00c (61.05)	153.76c (58.76)	143.71b (37.73)	131.29b (42.56)	176.24b (26.57)	1,117.88c (252.40)
P**		**<0.001**	**<0.001**	**<0.001**	**<0.001**	**<0.001**	**<0.001**	**<0.001**
**Region**								
North	15	95.60a (66.00)	110.20a (73.04)	38.27a (46.28)	76.47 (61.05)	35.00a (42.86)	87.47a (59.86)	443.00a (262.52)
Northeast	52	137.67a (90.07)	169.12ab (99.24)	70.98ab (70.69)	87.06 (54.17)	63.71a (48.85)	119.00ab (53.46)	647.54a (334.99)
Southeast	25	201.40b (110.47)	216.16b (102.99)	114.64b (88.07)	119.64 (62.43)	104.60b (64.38)	148.72b (62.14)	905.16b (457.31)
South	25	147.60ab (86.31)	189.64ab (84.06)	96.80ab (72.76)	89.32 (46.74)	72.00ab (47.64)	112.68ab (40.85)	708.04ab (296.81)
Central-West	9	123.00ab (97.48)	129.67ab (72.56)	53.00ab (65.28)	71.67 (46.09)	61.56ab (36.33)	98.00ab (34.07)	536.89ab (309.71)
P**		**0.008**	**0.006**	**0.009**	0.062	**0.001**	**0.006**	**0.001**
**Age**								
20–39	72	161.86a (93.78)	177.06 (98.75)	93.46a (76.89)	92.24 (53.56)	79.69a (52.01)	124.25 (56.15)	728.56a (366.18)
40–49	30	161.07a (100.45)	184.00 (98.31)	81.93ab (79.33)	98.10 (60.81)	82.50a (58.27)	122.67 (55.57)	730.27a (400.24)
50 or more	24	80.79b (66.47)	145.46 (87.98)	35.04b (42.87)	81.63 (58.25)	24.75ab (22.76)	95.46 (47.01)	463.13b (248.23)
P**		**0.002**	0.296	**0.004**	0.560	**<0.001**	0.075	**0.006**
**Sex**								
Male	10	152.60 (90.91)	143.50 (90.21)	55.60 (48.26)	101.50 (52.25)	81.70 (61.06)	106.10 (60.85)	641.00 (317.19)
Female	116	145.68 (96.50)	175.21 (97.41)	81.66 (76.83)	90.76 (56.49)	68.88 (53.40)	119.45 (54.76)	681.63 (373.43)
P**		0.827	0.323	0.295	0.563	0.473	0.465	0.739
**Time since graduation**								
Up to 10 years	35	162.97a (93.68)	171.26 (92.74)	91.31a (67.76)	96.40 (53.65)	74.43a (53.44)	118.69 (45.29)	715.06ab (335.83)
11–15 years	42	165.79a (96.77)	186.76 (108.06)	95.83a (85.58)	96.57 (54.04)	88.40a (52.26)	132.52 (59.63)	765.88a (397.33)
16–20 years	16	145.56ab (105.38)	175.31 (93.98)	83.63ab (87.79)	94.44 (58.06)	72.50ab (60.42)	120.81 (67.77)	692.25ab (435.82)
>20 years	33	103.91b (81.36)	155.03 (88.59)	44.52b (49.18)	78.85 (60.51)	40.27b (41.47)	98.91 (48.22)	521.48b (285.97)
P**		**0.024**	0,577	**0,016**	0,510	**0.001**	0.073	**0.031**
**Complementary training**								
Graduate certificate/Residency	59	137.41 (92.20)	166.12 (95.62)	75.93 (78.43)	91.97 (53.37)	69.14 (53.03)	119.25 (54.64)	659.81 (357.94)
Master’s degree	44	166.23 (101.10)	198.32 (98.58)	88.16 (68.27)	101.27 (59.98)	73.20 (54.40)	121.68 (58.34)	748.86 (380.26)
Doctorate/Post-Doctorate	23	130.61 (91.77)	140.52 (87.96)	72.57 (80.84)	72.22 (52.24)	65.52 (57.08)	109.87 (51.42)	591.30 (362.77)
P**		0.221	0.052	0.637	0.131	0.850	0.701	0.219
**Management training**								
Yes	56	113.41 (79.99)	150.14 (88.41)	46.98 (50.05)	76.39 (51.19)	52.20 (43.56)	104.00 (49.70)	543.13 (278.53)
No	70	172.49 (99.65)	190.73 (100.18)	105.67 (81.75)	103.79 (57.13)	84.06 (57.37)	129.90 (56.87)	786.63 (396.47)
P**		**<0.001**	**0.019**	**<0.001**	**0.006**	**0.001**	**0.008**	**<0.001**
**Time of service**								
Up to 10 years	43	148.86 (96.43)	162.19 (92.95)	81.33 (67.01)	90.14 (55.03)	70.70ab (53.32)	113.37 (49.11)	666.58 (349.93)
11–15 years	44	163.07 (99.88)	186.41 (109.48)	94.16 (87.98)	96.66 (53.61)	83.89a (52.48)	131.32 (58.27)	755.50 (406.28)
>20 years	39	124.33 (88.07)	168.79 (86.10)	61.23 (65.30)	87.54 (60.70)	53.23b (52.75)	109.33 (56.41)	604.46 (333.86)
P**		0.180	0.488	0.135	0.747	**0.034**	0.148	0.171
**Management experience time**								
<5 years	66	163.59a (95.53)	177.45 (96.00)	94.76a (77.66)	98.62 (54.11)	83.32a (56.98)	122.82 (56.05)	740.56a (374.14)
6–10 years	28	131.86ab (102.40)	154.96 (109.51)	67.43ab (77.25)	86.36 (55.77)	65.46ab (50.99)	111.32 (57.99)	617.39ab (400.09)
11 to 15 years	14	159.36ab (78.78)	209.00 (93.14)	87.36ab (78.88)	99.79 (64.18)	64.50ab (43.91)	129.71 (66.12)	749.71ab (342.71)
>=16 years	18	94.72b (81.48)	154.56 (77.96)	36.83b (33.47)	67.72 (54.01)	31.78b (32.31)	104.33 (34.25)	489.94b (235.16)
P**		**0.039**	0.299	**0.023**	0.184	**0.003**	0.454	**0.046**
**Time of experience in the role**								
<1 year	23	114.74 (72.14)	139.39 (99.72)	66.35 (76.69)	83.61 (50.36)	62.00 (46.72)	102.96 (62.29)	569.04 (332.87)
1 to 5 years	75	158.59 (98.97)	181.08 (96.04)	86.67 (75.65)	92.17 (56.51)	77.15 (57.40)	125.43 (53.22)	721.08 (375.71)
6 to 10 years	16	147.25 (110.03)	180.00 (106.61)	79.06 (86.86)	111.25 (61.20)	63.94 (51.41)	114.69 (65.40)	696.19 (423.47)
>=11 years	12	128.00 (88.65)	174.33 (79.87)	61.42 (50.69)	77.25 (55.79)	47.67 (42.27)	108.92 (30.49)	597.58 (284.30)
P**		0.245	0.341	0.561	0.361	0.254	0.330	0.304
**Number of bonds**								
1 bond	96	146.11 (97.90)	173.66 (95.21)	79.40 (75.93)	91.91 (56.74)	68.55 (54.61)	118.98 (54.15)	678.60 (373.37)
2 or 3 bonds	30	146.60 (90.06)	169.60 (103.73)	80.20 (73.90)	90.67 (54.71)	74.20 (52.19)	116.50 (59.05)	677.77 (357.84)
P**		0.981	0.842	0.959	0.916	0.618	0.831	0.991
**Type of bond**								
Public officer with job stability	74	165.86 (101.11)	196.45 (95.83)	90.38 (76.42)	101.16 (57.08)	75.80 (58.21)	128.20 (55.91)	757.85 (388.25)
CLT-hired	52	118.29 (80.48)	138.88 (88.79)	64.23 (71.25)	78.02 (52.11)	61.50 (46.32)	104.42 (51.34)	565.35 (307.47)
P**		**0.006**	**0.001**	0.054	**0.022**	0.128	**0.017**	**0.002**
**Weekly workload**								
Up to 40 hours	92	141.16 (95.02)	159.22 (92.66)	74.50 (71.28)	90.12 (57.18)	64.82 (52.28)	109.38 (53.23)	639.20 (356.53)
>=40 hours	34	159.94 (97.76)	209.15 (100.07)	93.35 (84.38)	95.65 (53.47)	83.65 (56.54)	142.76 (53.46)	784.50 (384.00)
P**		0.33	**0.01**	0.213	0.625	0.082	**0.002**	**0.049**

*t test for independent samples; **Analysis of variance model (ANOVA); Different letters represent statistically different means; Small (up to 199 beds), medium (200 to 399 beds) and large (over 400 beds) HUF size; Factor 1 = recognition, power, and professional identity; Factor 2 = safe and qualified care; Factor 3 = defense of values and rights; Factor 4 = working conditions; Factor 5 = ethical infractions; Factor 6 = work teams. Note: Variation of GMD score by factor, MDSN-Br (Brazilian Scale of Moral Distress in Nurses): F1 (0-396), F2 (0-396), F3 (0-288), F4 (0-216), F5 (0-216), F6 (0-252), GMD (0-1764). Source: the author.

## DISCUSSION

The causes of MD in nursing are varied and arise as a result of reciprocal relationships between individuals and organizations, where people and systems are connected^(11)^. Health care services, such as hospitals, have been studied as complex systems and pluralistic environments, in which power, legitimacy and authority are disseminated among managers, clinical professionals, regulatory agencies, among other parties, so that multilevel governance processes become the object of research and management experiences^(12)^. Leadership can also be approached as a multilevel process, present in the organization as a whole and not as an individual attribute; that is, a socially constructed process in everyday life, which includes communication, influence, adaptation, learning, power, and resistance^(13)^.

The precarious institutional ethical climate, characterized by representations such as less administrative support, less collaboration, fewer resources, and MD, generate dissatisfaction at work and increase nurses turnover^(14)^. The quantitative-qualitative studies carried out on DM show the participants’ characteristics with a special focus on the areas of hospital performance, complex care units, hemato-oncology, obstetric center, or adult or pediatric surgical clinics^(14,15,16,17,18)^. In the present study, large *HUF*s had the highest significant MD scores. Although the literature does not corroborate these findings, some investigations in large hospitals address issues such as stress^(19)^ or mental resources and work capacity of nursing workers^(20)^.

The variable lack of management training as a predictor of high-intensity MD in situations related to “Safe and qualified care”, “Working conditions” and “Work team” is characterized as an important result of this study. The theoretical bases acquired in the training are considered supporting elements of moral deliberation, favoring the development of ethical-moral competences and helping to overcome the barriers imposed in the MD process. Moral sensitivity is also taken as indispensable for a moral problem to be visible and become an object of ethical reflection^(1,21)^. Despite the need for studies that make more in-depth investigations of the relationship between MD and moral sensitivity, there is research that did not find such a relationship among nurses^(22)^.

The theme has multiple subjective variables and heterogeneous conditions. Ethical sensitivity is not only related to ethical knowledge and professional experience, but also to the hierarchical organizational climate, professional profile, attitude or behavior at work, and even the application of ethical knowledge in practice^(23,24)^.

Developing technical and ethical competence should be the target of management training, to add tacit knowledge of management to practical experience, assuming the co-responsibility of training for the incorporation of an ethical education agenda, supporting the exercise of moral agency, which prevents and confronts MD^(2,25)^.

In addition, it is important to note that “Safe and qualified care”, “Working conditions” and “Work team” are possible dimensions of primary (basilar) effort in nursing management practices, and on which *HUF* manager nurses showed greater anguish^(8)^. In the work process organization, the hospital nursing service has as its objects of managerial work the organization of work and nursing human resources, committed to providing better care^(26)^.

Regarding the professional bond nature, the nurse manager who has job stability (with longer working time in the *HUF*) had the highest rates of MD, especially in factors related to “Recognition, power and professional identity”, “Safe and qualified care”, “working conditions” and “Work team”. In most *HUF*/Ebserh, there is the coexistence of two types of bonds, those who have job stability and those CLT-hired (no stability), governed by different laws and rules, with the latter forms of contract occurring from 2013 onwards. International studies show that this reality is unique and difficult to adhere to. At the national level, a cross-sectional study carried out with 1,127 Brazilian nurses, using the MDSN-Br, also found that public servants with job stability showed the highest rates of MD, especially in factors related to “Safe and qualified care”, “Working conditions”, “Defense of values and rights” and “Work teams”^(27)^.

Among other professional characteristics, the highest rate of MD was observed among nurse managers conceived by this study as those with the least professional experience (younger, with less training time and management experience), showing a significant association in the general score and factors relevant to “Recognition, power, and professional identity”, “Defense of values and rights” and “Ethical infractions”. This experience can be synonymous with self-confidence, generated throughout the practice, and which possibly guides decision making, supported by intuition, knowledge, communication, use of (regulatory) standards, support and collaboration from experienced colleagues^(28)^.

More experienced nurses may have a greater degree of ethical sensitivity^(4)^, and in the same way, excel in cultivating moral resilience, developing ethical skills and competences that help them to connect with their primary intentions, thus reducing MD effects. On the other hand, longer experience can lead nurses to be exposed to continuous moral problems, accumulating (moral) residues of feelings of anguish and even generating moral sensitivity weakening (“desensitization”) and accommodation to MD^(27)^.

In fact, there are conflicting understandings and findings about the relationship between time of experience, sensitivity, and MD intensity. There is a study that corroborates this finding, highlighting that the longer nurse’s practice reduces suffering^(19)^; and there is research refuting this association^(29)^.

The variable related to the weekly workload of nurse managers showed a significant association with MD rate, especially in the factors that deal with “Safe and qualified care” and “Work team”. It is known that this characteristic is directly related to the intensity of work (tense and intense), which is a consequence of staff dimensioning, of versatility and flexibility of nursing practices; thus, as they compromise the quality of care, they produce embarrassment and dissatisfaction to the professional^(19)^.

The characteristics related to sex, complementary training, and number of bonds did not find a significant correspondence in the association with the MD rate, approaching the data found by another study^(18)^, but, opposed, in parts, by others^(27,29)^. Regarding these associations, there is no theoretical-practical clarification, and other empirical investigations are recommended. The existence of a greater tendency in the *HUFs* towards the construction of higher complementary training, as well as more possibilities of professional insertion in areas of teaching and research activities, due to the characteristics of their institutional nature, stands out.

Finally, regarding the approach to the MD determinants, it is mandatory to remember that the nurses who manage *HUFs* belong to complex organizational structures, in which they need to establish transparent and mutually supportive ethical relationships to mitigate MD complications and eliminate their sources, providing a healthy work environment, with the best conditions for care, teaching and research practices.

As limitations of the study, it is important to note that its results cannot be generalized and reflect HUF/Ebserh realities. In data collection process, it should be noted that the completion of the answers online was not directly observed, and it is based on the fidelity (personal and non-transferable aspect) of the reported data.

## CONCLUSION

Based on the present study, the association between sociodemographic and occupational variables and MD predictors in nursing managers at the *HUF* could be analyzed. It was found that nurses located in large *HUFs* experienced high levels of MD, with a significant association in all factors. In these complex structures, the design of an ethical climate can be essential to guide the dynamics of interactions between nurses and the organizational environment and the way they deal with ethical issues.

The lack of management training, with statistical significance in all factors, presented a high level of MD intensity in situations related to “Safe and qualified care”, “Working conditions” and “Work team”. The theoretical bases obtained in the training process articulated with ethical education are shown to be indispensable in the development of ethical-moral competences directed towards the management of teams and care itself.

The nurses who have job stability showed the highest rates of MD, especially in factors related to “Recognition, power and professional identity”, “Safe and qualified care”, “working conditions” and “Work team”. This aspect may be associated with the historical context of adhesion to Ebserh, with the increase in the plurality of institutional relations.

The highest MD rate was observed among nurse managers with the least professional experience, showing a significant association, especially in the factors relevant to “Recognition, power and professional identity”, “Defense of values and rights” and “Ethical infractions”. If, on the one hand, professional experience supports ethical sensitivity and moral deliberation, on the other hand, it can generate moral residue and ethical perception weakening, or even lead to excess self-confidence and inflexibility, which can inhibit sensitivity and even hinder the observation of the moral problem.

The weekly workload showed a significant association with MD rate, especially in the factors related to “Safe and qualified care” and “Work team”, a direct reflection of the intensity (load) of work present in the *HUFs*, reinforcing the historical nursing struggle for the regulation of the 30-hour workweek.

## ASSOCIATE EDITOR

Thiago da Silva Doming
